# The Relation Between Caregivers' Multiliterate Reading Habits and Their Children's Oral Health Status

**DOI:** 10.2196/ijmr.3210

**Published:** 2014-09-18

**Authors:** Divya S Parthasarathy, Susan M Bridges, Colman PJ McGrath, Terry KF Au, Hai Ming Wong, Cynthia KY Yiu

**Affiliations:** ^1^The University of Hong KongHong KongChina (Hong Kong); ^2^Centre for the Enhancement of Teaching and Learning/ Faculty of EducationThe University of Hong KongHong KongChina (Hong Kong); ^3^Prince Philip Dental HospitalDental Public HealthThe University of Hong KongHong KongChina (Hong Kong); ^4^PsychologyDepartment of PsychologyThe University of Hong KongHong KongChina (Hong Kong); ^5^Prince Philip Dental HospitalPaediatric DentistryThe University of Hong KongHong KongChina (Hong Kong)

**Keywords:** health literacy, multilingualism, multiliteracies, health informatics, medical consumerism, digital, caregiver, oral health status, paediatrics

## Abstract

**Background:**

Caregivers’ oral health literacy (OHL) assessment results have been found to be related to their children’s oral health status. A further aspect of this relationship may be the role of caregivers’ reading habits.

**Objective:**

Our goal was to describe the relationship between caregivers’ multimodal (digital and print) and multilingual (English and Chinese) reading habits, their OHL, and their child’s oral health status in Hong Kong.

**Methods:**

A random sample of 301 child-caregiver dyads was recruited from kindergartens in Hong Kong. Data included sociodemographic information and caregivers’ self-reported digital print and reading habits across two languages (Chinese and English). Caregivers’ OHL levels were assessed by two locally developed and validated oral health literacy assessment tasks: Hong Kong Rapid Estimate of Adult Literacy in Dentistry-30 (HKREALD-30) and the Hong Kong Oral Health Literacy Assessment Task for Pediatric Dentistry (HKOHLAT-P). Children’s oral health status was assessed using two measures: dental caries experience (number of decayed, missing, and filled teeth) and oral hygiene status (Visible Plaque Index).

**Results:**

Bivariate variations revealed significant differences in mean OHL scores between caregivers with different reading habits (*P*<.01). Correlations revealed significant associations between caregivers’ practices of reading multimodal (print/digital) and multilingual (English/Chinese) texts, their literacy levels, and their children’s oral health status (*P*<.01). Adjusting for sociodemographics and all other reading habits in the regression analysis, the caregivers' habit of reading digital and print texts was significantly retained in the final model. Regression analysis revealed significant associations between caregivers’ reading habits (digital Chinese) and their OHL word recognition scores: OR 5.00, 95% CI 1.10-3.65, *P*=.027. Significant associations were also evident for their OHL comprehension scores (digital Chinese: OR 2.30, 95% CI 1.30-4.20, *P*=.004; print Chinese: OR 2.50, 95% CI 1.40-4.30, *P*=.001). However, no significant associations were found between caregivers' reading habits and child’s oral health status (*P*>.05).

**Conclusions:**

Caregivers’ habits of reading print and digital Chinese texts are significantly associated with their OHL scores. Their reading habits, however, do not affect their children’s oral health status.

## Introduction

Health literacy is a concept that is both old and new [1]. One oft-cited definition from the World Health Organization (WHO) indicates it to be “a representation of the cognitive and social skills which determine the motivation and ability of individuals to gain access to, understand and use information in ways which promote and maintain good health” [2]. Early studies to develop efficient, pre-consultation literacy assessment tools [3,4] and patient education programs in English [5] noted the focus on functionalist aspects of health literacy to be a major limitation. Analogous critique has been leveled against early definitions of a more recent but associated concept—oral health literacy (OHL) [6-9]. While the functionalist studies to date have documented clear connections between caregiver health literacy and child health outcomes [10], as well as between caregiver oral health literacy (OHL) and child oral health outcomes [11-13], more work needs to be done to understand the connection between caregiver literacy and child health status. A second consideration is the critical re-thinking of what constitutes literacy in the modern world. There has been a shift in literate practices in recent years from traditional print texts to digital texts and multimodal forms (eg, hypertextual, audio-visual, gestural spatial). The combination of these new and old literate practices in highly diverse modern communities has given rise to the multiliteracies movement [14], which has examined the future of literacy and literacy pedagogy. The original multiliteracies design framework indicated how individuals engage with varying semiotic codes to identify, read, and create new texts [15].

In terms of implications for health literacy, research has shown that people with better digital and health knowledge can be expected to consume more information in various forms (digital and print texts) [16]. The field of medical informatics has developed content-specific, multiliterate practices as more individuals are relying on the Internet to access their (oral) health care-related information. While recent studies have emphasized the need for research explaining the use of information accessed online [17], work to date has found that, in general, people who seek online health information are more educated, earn more, and are more likely to have high-speed Internet access at home and at work [18,19]. Online health information is being accessed from various sources actively exchanging health information, including websites run by organizations, homepages run by individuals, online support groups, and blogs. A 2009 survey by Pew Internet and American Life Project found that approximately 61% of adults in the United States looked online for health information [20], and approximately 66% of health information seekers started with search engines such as Google or Yahoo with approximately 27% starting with specific health-related websites [21]. Other surveys have indicated that approximately 65% of participants searched for health information for at least half of the time they were online [22]. Together, these studies suggest that reading digital texts, especially via Internet searching, could reflect health information seeking behaviors.

The modern “multiliteracies” view, which rethinks the nature of texts, has also considered issues of diversity where individuals know or use more than one language system even if they do not live in a multilingual community [23,24]. Bilingual education and biliteracy research [25-27] indicate that bilinguals vary considerably in their command and usage of their two languages [28]. In addition to language usage, bilinguals may also vary in their cultural identity and various social variables. Hong Kong is a case in point. With Hong Kong’s policy of trilingualism and close relationship with China, Mandarin and English are learned and used in the territory; however, the vast majority of citizens are ethnic Chinese who speak Chinese (Cantonese dialect) as their native and dominant language. The longstanding practice of using English as a medium of instruction in secondary and tertiary education means that most educated Hong Kong citizens are fluent readers—even if not fluent speakers—of English.

English is an especially interesting example of a second or an additional language. Because the majority of advances in science and technology during the 20^th^ century were published in English, it has therefore become the common language of science and technology. Studies exploring the relation between bilingualism or multilingualism, multimodality, and health outcomes are rare. Therefore, this study examines caregivers’ multilingual and multimodal literacy—especially involving English as their common language for medical and oral health knowledge—and its relation to their children’s oral health. Given that the field of OHL has begun developing instruments in non-English contexts, such as Spanish [29] and Chinese [30-32], further examination is warranted. Despite the documented links between (oral) health literacy and (oral) health outcomes, as well as those between parental OHL and child oral health status [11-13], little is known about whether caregivers’ OHL levels and their reading habits can make a difference to their children’s oral health. This study responded to this research gap by examining the relation between (1) caregivers’ multimodal and multilingual reading habits, (2) their OHL scores, and (3) the oral health status of their preschool children in Hong Kong.

## Methods

### Sample Recruitment

A random sample of 301 preschool child-caregiver dyads living in Hong Kong participated. Among the 316 dyads recruited, 301 completed assessments; the response rate was 95.3%. The sample frame consisted of children from 10 kindergartens on Hong Kong Island (each with an enrollment of 70 children or more). One in four kindergartens was randomly selected and within each kindergarten, children were randomly selected for recruitment. All Chinese children aged 5 years who attended grade three (K3) in these 10 kindergartens were chosen randomly (by random digit tables). Their parents were contacted through the kindergartens with an invitation letter explaining the objectives of the project, and the consent form was distributed. Participation was voluntary, and no additional efforts were made to enroll the subjects. Eligibility criteria included healthy children who (1) were 5 years of age, and (2) were accompanied by a primary caregiver who could speak Cantonese and read traditional Chinese script. Children with specific learning disabilities or requiring learning support, and caregivers who could not read and write Cantonese were excluded from the study.

Using SAS software version 9.3, sample power was calculated based on Fisher’s Z test for Pearson correlation to have a 90% chance with two-sided test at a 5% significant level for detecting at least a 0.2 correlation; therefore 258 parent-child dyads would be sufficient. Allowing for potential non-response of about 15%, 316 dyads were recruited.

This study was approved by the by the Institutional Review Board of the University of Hong Kong/Hospital Authority Hong Kong West Cluster (HKU/HA HKW IRB) (Ref: UW 09-184).

### Data Collection

On arrival, dyads were assigned identifiers, and caregivers completed questionnaires (comprising pre-test background questions on family sociodemographics and caregiver self-reported reading habits) and underwent OHL assessments. Their children underwent clinical examinations of oral health status; assessments were conducted simultaneously and the assessors were kept blind of other assessors’ data. OHL assessment began with a word-recognition test using the Hong Kong Rapid Estimate of Adult Literacy in Dentistry-30 (HKREALD-30). Each caregiver was asked to read aloud a list of Chinese words related to oral health (eg, labels of parts in the mouth, dental procedures). It was conducted as an interview by trained and calibrated examiners and took about 2 minutes [30]. Immediately afterwards, a comprehension literacy assessment using the Hong Kong Oral Health Literacy Assessment Task-Pediatric Dentistry (HKOHLAT-P) was administered to the caregivers. This paper-and-pen assessment took about 45 minutes. It consists of 3 sections: (1) oral health knowledge section, (2) oral health-related numeracy, and (3) oral health-related reading comprehension. The scores of HKOHLAT-P range from 0-52 [31,32].

Children’s oral health status was assessed by trained and calibrated examiners, using the methods and criteria as prescribed by the WHO basic oral health survey protocol [33]. This included an assessment of experience with dental caries—number of decayed, missing, and filled teeth (dmft). The Visible Plaque Index (VPI) [34] was used to assess oral hygiene status of the children by recording plaque deposits for various sites around the tooth to provide a summary score of oral hygiene—number of sites with dental plaque divided by number of sites examined.

### Statistical Analyses

The data analysis was carried out using the PASW (Predictive Analytics Software) statistics 18.0. Descriptive statistics were produced to examine the profile of the study group. Bivariate analyses examined variations between caregivers’ reading habits and their literacy levels (Table 1). Correlation analysis (Spearman correlation) between the two literacy instruments was conducted and was also conducted between the caregiver’s multilingual reading habits, caregiver’s habit of reading multimodal, multilingual texts, and child’s oral health status (Table 2).

Multiple logistic regression analysis was carried out with the two OHL assessment instruments as the dependent variables (Tables 5.1 and 5.2 in Multimedia Appendix 1) and the independent variables being caregiver, child sociodemographics and the caregiver’s four main reading habits (print and digital Chinese; print and digital English) in 6 separate models (Models 1-5: unadjusted models—Model 1: Sociodemographics; Models 2-5: Sociodemographics, and one reading habit in each model respectively; Model 6: adjusted model with all independent variables). Similar analyses were also performed with two measures of the child’s oral health status (dmft and VPI) as dependent variables (Tables 5.3 and 5.4 in Multimedia Appendix 1).

**Table 1:** Results (independent sample *t* test) showing bivariate variations between caregivers’ reading habits and their OHL test scores (n=301 dyads).

Reading materials		HKREALD-30		HKOHLAT-P	
		Mean (SD)	*P* value	Mean (SD)	*P* value
**Print Chinese**					
	None	16.1 (3.28)	<.001^a^	33.0 (7.52)	<.001^a^
	<1 hour	22.13 (3.78)		41.3 (6.44)	
	1-3 hours	23.3 (4.35)		43.5 (4.67)	
	>3 hours	23.9 (3.02)		44.8 (3.96)	
**Print English**					
	None	20.5 (3.92)	<.001^a^	41.1 (6.65)	.005^b^
	< 1 hour	23.3 (3.74)		43.0 (5.86)	
	1-3 hours	23.6 (4.36)		43.9 (4.36)	
	>3 hours	24.4 (2.69)		44.4 (4.22)	
**Digital Chinese**					
	None	20.1 (4.21)	<.001^a^	38.9 (6.80)	<.001^a^
	< 1 hour	21.2 (3.92)		41.4 (6.28)	
	1-3 hours	23.7 (4.35)		43.8 (5.01)	
	>3 hours	23.9 (3.02)		44.2 (4.59)	
**Digital English**					
	None	21.0 (3.87)	<.001^a^	40.9 (6.22)	<.001^a^
	<1 hour	22.8 (4.32)		42.3 (6.42)	
	1-3 hours	24.0 (3.67)		44.2 (4.18)	
	>3 hours	24.1 (3.08)		44.8 (3.98)	
**Factual Chinese**					
	None	17.1 (4.67)	.001^b^	36.7 (10.7)	<.001^a^
	<1 hour	22.7 (3.93)		40.9 (5.84)	
	1-3 hours	23.4 (3.59)		44.1 (4.97)	
	>3 hours	22.9 (4.14)		43.5 (5.06)	
**Factual English**					
	None	21.8 (4.42)	.191	42.6 (6.91)	.194
	<1 hour	23.0 (3.89)		42.5 (5.68)	
	1-3 hours	23.4 (3.46)		44.6 (5.02)	
	>3 hours	22.6(4.53)		43.0 (5.12)	
**Creative Chinese**					
	None	20.7 (5.56)	.193	40.6 (8.06)	.205
	<1 hour	22.7 (3.92)		42.4 (5.65)	
	1-3 hours	22.9 (4.11)		43.4 (5.54)	
	>3 hours	23.5 (3.52)		43.7 (5.16)	
**Creative English**					
	None	21.6 (4.34)	.041^c^	42.6 (6.55)	.417
	<1 hour	23.1 (3.72)		42.5 (5.58)	
	1-3 hours	23.3 (4.05)		43.8 (5.08)	
	>3 hours	23.6 (3.74)		43.3 (5.21)	

^a^ *P*<.001.

^b^ *P*<.01.

^c^ *P*<.05.

**Table 2:** Correlations (Spearman correlation coefficient) between caregivers’ reading habits (multimodal, multilingual) and their child’s oral health status.

Reading habits			dmft		VPI	
			*r*	*P* value	*r*	*P* value
**Hours spent reading digital texts**			-.280	<.001^a^	-.068	.240
	**Multilingual texts**					
		Digital Chinese texts	-.230	<.001^a^	-.064	.270
		Digital English texts	-.270	<.001^a^	-.095	.102
**Reading habits scale**			-.239	<.001 ^a^	-.065	.260
	**Multilingual texts**					
		Chinese texts	-.191	<.001^a^	-.042	.464
		English texts	-.234	<.001^a^	-.085	.140

^a^Significant at *P*<.001.

## Results

The sociodemographic profile of the participants is presented in Table 3. Three quarters of the children (75.4%, 227/301) had a dental caries experience ((dmft>0) and mean dmft was 4.2 (SD 4.5; see Table 4). Most of the dental caries experience was related to untreated dental decay: the prevalence of decayed teeth (dt) was 68.8% (207/301) and the mean dt was 3.3 (SD 3.9). Almost all children had evidence of plaque deposits at one or more sites (99.3%, 299/301), and the mean VPI was 63.5 (SD 20.4).

The mean of caregivers’ multilingual reading habits in Chinese and English were 8.00 (SD 2.81, range 0.00-12.00) and 5.73 (SD 3.34, range 0.00-12.00) respectively. The mean HKREALD-30 score was 23.0 (SD 3.97, range 9.00-30.00), and the mean HKOHLAT-P score was 43.6 (SD 5.59, range 21.00-52.00).

Bivariate variations performed between caregivers’ reading habits and their literacy levels revealed systematic variations in their means (Table 1). Caregivers’ practice of reading print Chinese and English, digital Chinese and English, and factual Chinese texts were significantly associated with their own OHL test scores (*P*<.001). Associations were also found between reading factual English texts and their OHL word recognition scores (*P*=.041).

Importantly, correlations indicated a significant although modest association between the children’s dmft and the caregivers’ reading habits in English (*r*=-.234, *P*<.001), as well as the caregivers’ Chinese reading habits (*r*=-.191, *P*=.001) (Table 2). Correlations also indicated significant although modest associations between children’s dmft and their caregivers’ habit of reading digital texts in English (*r*=-.230, *P*<.001) and Chinese (*r*=-.270, *P*<.001). However, no analogous associations were found for the children’s VPI (*P*>.05) (Table 4).

To further understand these correlations, multiple logistic regression analyses were performed (see Multimedia Appendix 1). The analyses indicated that the caregivers who read more digital Chinese texts were more likely to score better in the OHL word recognition test: OR 2.00, CI 1.10-3.65, *P*=.027 (see Table 5.1, Model 3, in Multimedia Appendix 1). After adjusting for sociodemographics and all other reading habits, caregivers’ habit of reading digital texts was significantly retained in the final model: OR 2.00, CI 1.10-3.65, *P*=.027 (see Table 5.1, Model 6, in Multimedia Appendix 1). Further analyses with HKOHLAT-P (Table 5.2 in Multimedia Appendix 1) also indicated that caregivers’ habit of reading print Chinese (OR 2.50, CI 1.40-4.30, *P*=.001) and digital Chinese texts (OR 2.30, CI1.30-4.20, *P*=.004) were associated with an increased likelihood of having a higher score in their comprehension test (see Table 5.2, Models 2 and 3, in Multimedia Appendix 1). After adjusting for sociodemographics and all the other reading habits, caregivers’ habit of reading print Chinese texts was significantly retained in the final model: OR 2.50, CI 1.40-4.30, *P*=.001 (see Table 5.1, Model 6, in Multimedia Appendix 1).

Multiple regression analysis with child’s oral health status as a dependent variable, however, revealed that except for the education level of the caregiver (dmft model: OR 0.40, CI 0.20-0.65, *P*<.001; Table 5.3 in Multimedia Appendix 1; VPI model: OR 0.60, CI 0.40-0.90, *P*=.028; Table 5.4 in Multimedia Appendix 1), none of the reading habit variables were retained in the final caries and VPI models in both adjusted and unadjusted analyses (Table 5.3 in Multimedia Appendix 1), indicating that caregivers’ education level is by far the strongest predictor of child’s oral health status.

**Table 3:** Profile of the study population (caregivers and children) (n=301 dyads).

Characteristics			n (%)
**Caregiver**			
	**Gender**		
		Mother	223 (74.1)
		Father/ other caregiver	78 (25.9)
	**Educational level**		
		Secondary school or lower	155 (51.5)
		Above secondary school	146 (48.5)
	**Age, years**		
		<40	192 (63.8)
		≥40	109 (36.2)
	**Income level** ^a^ **, HKD**		
		< 20,000	102 (33.9)
		≥ 20,000	199 (66.1)
**Child**			
	**Gender**		
		Male	134 (44.5)
		Female	167 (55.5)

^a^20,000 HKD=US $2580

**Table 4:** Clinical oral health status of children: dental caries experience (dmft) and oral hygiene status (VPI) (n=301 dyads).

Clinical oral health status		%	n	Mean	SD	Minimum	Maximum
**Dental caries experience**							
	dmft^a^	75.4	227^a^	4.2	4.5	0.0	20.0
	decayed teeth (dt)	68.8	207^a^	3.3	3.9	0.0	18.0
	missing teeth (mt)	30.2	91^a^	0.7	1.3	0.0	6.0
	filled teeth (ft)	7.6	23^a^	0.2	0.9	0.0	9.0
**Oral hygiene status**							
	VPI^b^	99.3	299^b^	63.5	20.4	0.0	100

^a^dmft>0

^b^VPI>0

## Discussion

### Principal Results

This study indicates significant associations between caregivers’ reading habits and their OHL. The main hypotheses tested were that an individual who spends more hours reading texts (both Chinese and English) should (1) perform better in a print-based OHL test, and (2) have children with better oral health.

### Strengths and Limitations

The results presented here should be considered in light of the study’s limitations. First, the study used a cross-sectional design, making it difficult to draw causal inferences. Second, the data were collected from socioeconomic background neighborhoods higher than that in Hong Kong as a whole (Table 3); this sample might not be representative of the entire population of preschool parent-child dyads living in other parts of the Hong Kong Special Administrative Region (HKSAR). Since correlations in the present study were weak but significant, further studies with much larger and diverse samples are required to produce stronger correlations. Finally, the developed instruments focus only on the functional OHL of the caregivers [35]; future research should also focus on other theoretically important dimensions such as communicative literacy to higher levels of critical health literacy [1,36]. Furthermore, since the instruments were developed in traditional Chinese script and Cantonese vocabulary, care should be taken in extrapolating these instruments to other Chinese dialects such as Mandarin. Future studies should evaluate these instruments in more diverse populations.

Despite these limitations, the study has several strengths. First, this is multidisciplinary research involving investigators from literacy, psychology, dental public health, and pediatric dentistry collaborating together to address a multifaceted issue. The use of locally developed, validated instruments to measure OHL levels is another important strength. Third, dmft and VPI were used to assess the dental disease severity of each child. By contrast, all other known OHL studies have examined disease severity by using parental oral health status reports or chart reviews [8,37]. Fourth, trained calibrated examiners interviewed caregivers as well as performed the clinical examinations.

### Comparison With Prior Work

Currently, about 60% of Hong Kong adults find online health information to be useful [38]. Our study constitutes a first step in exploring factors such as caregivers’ multimodal reading habits (print and digital texts) and their OHL scores in influencing their children’s oral health status. The present findings are likely to draw more attention to the field of medical informatics in China. With more patients relying on the Internet as their information source prior to medical consultations [39], there has been a shift in traditional doctor-patient relationships [17]. Indeed, approximately 80% of physicians reported in a 2011 study that patients presented printed Internet-sourced health information during their clinic visits [40]. Interest in the Internet as a communication tool for health-related information is also on the rise [41].

People who seek online health information are typically patients or their friends/relatives [42], with various goals and levels of Internet search experience [43]. In general, women are more likely than men to search for health information [44]. Given that approximately 75% of caregivers in our study were female, our findings offer a valuable window on the possible relations between caregivers’ reading habits and health-information seeking. Studies in general medicine have shown people’s satisfaction in seeking health information online [41] and have shown that sicker patients approached their doctors with more information accessed online [45]. These indicate possible associations between patients’ accessing of digital texts and their health status. The correlations uncovered by the present study found a significant, albeit modest, association between caregivers’ reading habits (multimodal, multilingual) and their OHL scores and their children’s dmft (decayed, missing, or filled teeth status) status. However, the caregivers’ self-reported reading habits in this study were not explicitly restricted to hours spent on reading online (oral) health information. Future studies would benefit from deeper investigation of this aspect.

The logistic regression analyses suggested that only sociodemographics such as education, income, and the multimodal reading habits (digital Chinese for word recognition scores and print Chinese and digital Chinese for reading comprehension) were predictors of caregivers’ OHL test scores. Note that HKREALD-30, one of our OHL measures, was developed from a keyword corpus database of locally available public health materials, including materials from online sources such as government oral health promotion websites. Additionally, this word-recognition task presents words in isolation selected for their level of frequency and so are likely to occur across multiple modalities (print and Web-based). It makes sense that the caregivers’ reading habits predicted their HKREALD-30 scores.

HKOHLAT-P, the other OHL measure, assesses reading skills and not just word recognition. It is language rich and was generated from both print and digital texts [32]. It therefore makes sense that reading print Chinese and digital Chinese were significantly retained in the final unadjusted models, and print Chinese was retained along with the income level of the caregiver in the final adjusted model (Table 5.2 in Multimedia Appendix 1). Associations between multilingual reading habits and OHL test scores and associations between multimodal, multilingual reading habits of caregivers and their children’s oral health status were not evident in the final models in the present study, suggesting further research is needed to understand these issues better.

### Conclusions

This study suggested that caregivers’ habits of reading print and digital text were significantly associated with their OHL scores, although no associations were found between caregivers’ reading habits and their children’s oral health status. The study in OHL among a Chinese-speaking community (Hong Kong) reported here supports a widening of the definition of health literacy by highlighting the importance of health informatics, especially for oral health promotion in a multilingual territory such as Hong Kong.

## Multimedia Appendix 1

Results of multiple regression analysis.

## Abbreviations

dmft: decayed, missing, filled teeth
HKD: Hong Kong Dollars
HKSAR: Hong Kong Special Administrative Region
HKOHLAT-P: Hong Kong Oral Health Literacy Assessment Task-Pediatric Dentistry
HKREALD-30: Hong Kong Rapid Estimate of Adult Literacy in Dentistry-30
OHL: oral health literacy
PASW: Predictive Analytical Software
VPI: Visible Plaque Index
WHO: World Health Organization
