# Supplementary material for: The Relation Between Caregivers' Multiliterate Reading Habits and Their Children's Oral Health Status
Source: Interact J Med Res. 2014 Sep 18;3(3):e13. doi: 10.2196/ijmr.3210 (PMC4180347; doi:10.2196/ijmr.3210)
Supplement: Supplementary file 1 [file ijmr_v3i3e13_app1.pdf]

Multimedia Appendix 1. Results of multiple regression analysis.

Table 5.1. Results of multiple regression analysis.

MODEL-1 (HKREALD-30) [d]

**Variable**	**B**	**SE**	**OR**	**95%CI**	***P*-value**
Education	0.70	0.30	2.00	(1.20-3.55)	0.013 [c]
Income	1.60	0.30	5.00	(2.70-9.30)	<0.001 [a]

MODEL-2 (HKREALD-30) [d]

**Variable**	**B**	**SE**	**OR**	**95%CI**	***P*-value**
Education	0.70	0.30	2.00	(1.20-3.55)	0.013 [c]
Income	1.60	0.30	5.00	(2.70-9.30)	<0.001 [a]

MODEL-3 (HKREALD-30) [d]

**Variable**	**B**	**SE**	**OR**	**95%CI**	***P*-value**
Education	0.60	0.30	1.80	(1.00-3.20)	0.044 [c]
Income	1.50	0.30	4.50	(2.40-8.40)	<0.001 [a]
Digital Chinese	0.70	0.30	2.00	(1.10-3.65)	0.027 [c]

MODEL-4 (HKREALD-30) [d]

**Variable**	**B**	**SE**	**OR**	**95%CI**	***P*-value**
Education	0.70	0.30	2.00	(1.20-3.55)	0.013 [c]
Income	1.60	0.30	5.00	(2.70-9.30)	<0.001 [a]

MODEL-5 (HKREALD-30) [d]

**Variable**	**B**	**SE**	**OR**	**95%CI**	***P*-value**
Education	0.70	0.30	2.00	(1.20-3.55)	0.013 [c]
Income	1.60	0.30	5.00	(2.70-9.30)	<0.001 [a]

MODEL-6 (HKREALD-30) [d]

**Variable**	**B**	**SE**	**OR**	**95%CI**	***P*-value**
Education	0.60	0.30	1.80	(1.00-3.20)	0.044 [c]
Income	1.50	0.30	4.50	(2.40-8.40)	<0.001 [a]
Digital Chinese	0.70	0.30	2.00	(1.10-3.65)	0.027 [c]

Significant at [a]*P*<.001, [b]*P*<.01, [c]*P*<.05, [d]-(HKREALD-30-50% Cutoff point)

(Models 1-5: unadjusted models, Model 6: adjusted model)

Model 1: Dependent variable: HKREALD-30: Independent variables: caregiver’s and child’s sociodemographics
Model 2: Dependent variable: HKREALD-30, Independent variables: caregiver’s and child’s sociodemographics, caregiver’s reading habits (Print Chinese)
Model 3: Dependent variable: HKREALD-30, Independent variables: caregiver’s and child’s sociodemographics, caregiver’s reading habits (Digital Chinese)
Model 4: Dependent variable: HKREALD-30, Independent variables: caregiver’s and child’s sociodemographics, caregiver’s reading habits (Print English)
Model 5: Dependent variable: HKREALD-30, Independent variables: caregiver’s and child’s sociodemographics, caregiver’s reading habits (Digital English)
Model 6: Dependent variable: Adjusted model: HKREALD-30, Independent variables: caregiver’s and child’s sociodemographics, caregiver’s reading habits (Print Chinese, Digital Chinese, Print English and Digital English

Table 5.2. Results of multiple regression analysis.

MODEL-1 (HKOHLAT-P) [d]

**Variable**	**B**	**SE**	**OR**	**95%CI**	***P*-value**

MODEL-2 (HKOHLAT-P) [d]

**Variable**	**B**	**SE**	**OR**	**95%CI**	***P*-value**

Variable	B	SE	OR (95%CI)	*P*-value
Income	1.25	0.30	3.40 (1.85-6.30)	<0.001[a]

Variable	B	SE	OR (95%CI)	*P*-value
Income	1.35	0.30	3.85 (2.20-6.70)	<0.001[a]
Print Chinese	0.90	0.30	2.50 (1.40-4.30)	0.001[b]

MODEL-3 (HKOHLAT-P) [d]

Variable	B	SE	OR 95%CI	*P*-value
Income	1.30	0.30	3.70 (2.10-6.45)	<0.001[a]
Digital Chinese	0.85	0.30	2.30 (1.30-4.20)	0.004[b]

MODEL-4 (HKOHLAT-P) [d]

Variable	B	SE	OR 95%CI	*P*-value
Income	1.25	0.30	3.40 (1.85-6.30)	<0.001[a]

MODEL-5 (HKOHLAT-P) [d]

Variable	B	SE	OR 95%CI	*P*-value
Income	1.25	0.30	3.40 (1.85-6.30)	<0.001[a]

MODEL-6 (HKOHLAT-P) [d]

Variable	B	SE	OR 95%CI	*P*-value
Income	1.30	0.30	3.75 (2.15-6.50)	<0.001[a]
Print Chinese	0.90	0.30	2.50 (1.40-4.30)	0.001[b]

Significant at [a]*P*<.001, [b]*P*<.01, [c]*P*<.05, [d] - HKOHLAT-P-50% Cutoff point

(Models 1-5: unadjusted models, Model 6: adjusted model)

Model 1: Dependent variable: HKOHLAT-P: Independent variables: caregiver's and child's sociodemographics
Model 2: Dependent variable: HKOHLAT-P, Independent variables: caregiver's and child's sociodemographics, caregiver's reading habits (Print Chinese)
Model 3: Dependent variable: HKOHLAT-P, Independent variables: caregiver's and child's sociodemographics, caregiver's reading habits (Digital Chinese)
Model 4: Dependent variable: HKOHLAT-P, Independent variables: caregiver's and child's sociodemographics, caregiver's reading habits (Print English)
Model 5: Dependent variable: HKOHLAT-P, Independent variables: caregiver's and child's sociodemographics, caregiver's reading habits (Digital English)
Model 6: Dependent variable: Adjusted model: HKOHLAT-P, Independent variables: caregiver's and child's sociodemographics, caregiver's reading habits (Print Chinese, Digital Chinese, Print English and Digital English)

Table 5.3. Results of multiple regression analysis

MODEL-1 (dmft)

Variable	B	SE	OR	95%CI	*P*-value
Education	-0.95	0.30	0.40	(0.20-0.65)	<0.001[a]

MODEL-2 (dmft)

Variable	B	SE	OR	95%CI	*P*-value
Education	-0.95	0.30	0.40	(0.20-0.65)	<0.001[a]

MODEL-3 (dmft)					
**Variable**	**B**	**SE**	**OR**	**95%CI**	***P*-value**
Education	-0.95	0.30	0.40	(0.20-0.65)	<0.001[a]

MODEL-4 (dmft)					
**Variable**	**B**	**SE**	**OR**	**95%CI**	***P*-value**
Education	-0.95	0.30	0.40	(0.20-0.65)	<0.001[a]

MODEL-5 (dmft)					
**Variable**	**B**	**SE**	**OR**	**95%CI**	***P*-value**
Education	-0.95	0.30	0.40	(0.20-0.65)	<0.001[a]

MODEL-6 (dmft)					
**Variable**	**B**	**SE**	**OR**	**95%CI**	***P*-value**
Education	-0.95	0.30	0.40	(0.20-0.65)	<0.001[a]

Significant at [a]*P*<.001, [b]*P*<.01, [c]*P*<.05

(Models 1-5: unadjusted models, Model 6: adjusted model)

Model 1: Dependent variable: dmft: Independent variables: caregiver's and child's sociodemographics.
Model 2: Dependent variable: dmft, Independent variables: caregiver's and child's sociodemographics, caregiver's reading habits (Print Chinese)
Model 3: Dependent variable: dmft, Independent variables: caregiver's and child's sociodemographics, caregiver's reading habits (Digital Chinese)
Model 4: Dependent variable: dmft, Independent variables: caregiver's and child's sociodemographics, caregiver's reading habits (Print English)
Model 5: Dependent variable: dmft, Independent variables: caregiver's and child's sociodemographics, caregiver's reading habits (Digital English)
Model 6: Adjusted model: Dependent variable: dmft, Independent variables: caregiver's and child's sociodemographics, caregiver's reading habits (Print Chinese, Digital Chinese, Print English and Digital English)

Table 5.4 Results of multiple regression analysis.

MODEL-1 (VPI)						MODEL-2 (VPI)					
**Variable**	**B**	**SE**	**OR**	**95%CI**	***P*-value**	**Variable**	**B**	**SE**	**OR**	**95%CI**	***P*-value**
Education	-0.50	0.20	0.60	(0.40-0.90)	0.028[c]	Education	-0.50	0.20	0.60	(0.40-0.90)	0.028[c]

MODEL-3 (VPI)						MODEL-4 (VPI)					
**Variable**	**B**	**SE**	**OR**	**95%CI**	***P*-value**	**Variable**	**B**	**SE**	**OR**	**95%CI**	***P*-value**
Education	-0.50	0.20	0.60	(0.40-0.90)	0.028[c]	Education	-0.50	0.20	0.60	(0.40-0.90)	0.028[c]

MODEL-5 (VPI)						MODEL-6 (VPI)					
**Variable**	**B**	**SE**	**OR**	**95%CI**	***P*-value**	**Variable**	**B**	**SE**	**OR**	**95%CI**	***P*-value**
Education	-0.50	0.20	0.60	(0.40-0.90)	0.028[c]	Education	-0.50	0.20	0.60	(0.40-0.90)	0.028[c]

Significant at [a]*P*<.001, [b]*P*<.01, [c]*P*<.05

(Models 1-5: unadjusted models, Model 6: adjusted model)

Model 1: Dependent variable: VPI (median 65%): Independent variables: caregiver's and child's sociodemographics.
Model 2: Dependent variable: VPI (median 65%): Independent variables: caregiver's and child's sociodemographics, caregiver's reading habits (Print Chinese)
Model 3: Dependent variable: VPI (median 65%): Independent variables: caregiver's and child's sociodemographics, caregiver's reading habits (Digital Chinese)
Model 4: Dependent variable: VPI (median 65%): Independent variables: caregiver's and child's sociodemographics, caregiver's reading habits (Print English)
Model 5: Dependent variable: VPI (median 65%): Independent variables: caregiver's and child's sociodemographics, caregiver's reading habits (Digital English)
Model 6: Dependent variable: Adjusted model: VPI (median 65%), Independent variables: caregiver's and child's sociodemographics, caregiver's reading habits (Print Chinese, Digital Chinese, Print English and Digital English)
